# On the core bacterial flora of *Ixodes persulcatus* (Taiga tick)

**DOI:** 10.1371/journal.pone.0180150

**Published:** 2017-07-10

**Authors:** Shuo Sui, Yu Yang, Yi Sun, Xumin Wang, Guoliang Wang, Guangle Shan, Jiancheng Wang, Jun Yu

**Affiliations:** 1 CAS Key Laboratory of Genome Sciences and Information, Beijing Institute of Genomics, Chinese Academy of Sciences, Beijing, China; 2 University of Chinese Academy of Sciences, Beijing, China; 3 Chinese Academy of Inspection and Quarantine, Beijing, China; 4 Beijing Institute of Microbiology and Epidemiology, Beijing, China; Jilin University, CHINA

## Abstract

*Ixodes persulcatus* is a predominant hard tick species that transmits a wide range of human and animal pathogens. Since bacterial flora of the tick dwelling in the wild always vary according to their hosts and the environment, it is highly desirable that species-associated microbiomes are fully determined by using next-generation sequencing and based on comparative metagenomics. Here, we examine such metagenomic changes of *I*. *persulcatus* starting with samples collected from the wild ticks and followed by the reared animals under pathogen-free laboratory conditions over multiple generations. Based on high-coverage genomic sequences from three experimental groups–wild, reared for a single generation or R1, and reared for eight generations or R8 –we identify the core bacterial flora of *I*. *persulcatus*, which contains 70 species that belong to 69 genera of 8 phyla; such a core is from the R8 group, which is reduced from 4625 species belonging to 1153 genera of 29 phyla in the wild group. Our study provides a novel example of tick core bacterial flora acquired based on wild-to-reared comparison, which paves a way for future research on tick metagenomics and tick-borne disease pandemics.

## Introduction

The tick, *Ixodes persulcatus*, is a predominant hard tick species found in Europe, central and northern Asia, China, and Japan. Tick is the second most widely recognized transmission vectors of human diseases worldwide, second only to mosquitoes [1,2]. Ticks carry a large number of pathogens, including viruses, bacteria, fungi, and protozoa, which are transmitted among animals and humans, and among them, *Borrelia burgdorferi* and *Anaplasma phagocytophilum* cause Lyme disease [3,4] and granulocytic anaplasmosis [5], respectively. In addition, *I*. *persulcatus* is a vector for the tick-borne encephalitis virus [2], and together with *Babesia* [6, 7] and Rickettsiae, they cause spotted fever [8, 9].

As progresses made through various microbial sequencing programs, such as the Human Microbiome Project [10], we have learnt that symbiotic microorganisms play important roles in host growth, development, metabolism, and immune system [11]. Therefore, understanding microbial communities of a host and their functional components has become key objectives for species-based genomics. High-throughput sequencing technology has led to the idea and technology of metagenomics, and based on whole genome and 16s rRNA sequencing, ample metagenomic data have been put forward for many symbiont bacterial species [12–14], including those of the tick [15] and its variability in life cycle [16]; some studies have focused on metagenomic changes among different tick species [17], between the two sexes [18, 19], geographies [20, 21], and before-and-after meal [22]. However, it still remains unknown that how tick-borne metagenomes vary among hosts and under pathogen-free rearing conditions, especially in the definition of species-associated core microbial/bacterial flora [11, 13, 14, 23, 24].

To explore such core symbiotic bacterial flora of *I*. *persulcatus*, we start from wild *I*. *persulcatus*, rear the tick in specific pathogen-free (SPF) mice in a sterile environment, and examine its flora over multiple generations. We then use a next-generation-sequencing method, acquire high-coverage metagenomic data, and analyze them by comparing the metagenome change among the samples. Here, we report our results for defining the core bacteril flora of *I*. *persulcatus*.

## Materials and methods

### Tick collection, breeding, and storage

The *I*. *persulcatus* strain Sfh has been reared in our laboratory; it is originally from female ticks collected with cloth-dragging methods from a forest region of Suifenhe City (E = 131.17, N = 44.38), Heilongjiang Province of northeastern China. The natural enzootic cycle is completed by growing larvae and nymphs on shaved skin of SPF Balb/C mice. We used female adults for all experiments. Larvae were obtained by placing 1.0 g hatched eggs onto feeding patches glued to the shaved patches of SPF Balb/C mice. After feeding, all larvae were removed before they molted to the nymphal stage. Nymphs were molted to the adult stage (first generation), and some adult ticks were maintained at room temperature for 1–3 weeks prior to hatching for propagation to the eighth generation. Other adult ticks were frozen at −80°C for DNA extraction.

### DNA preparation and sequencing

Before DNA extraction, 30 to 40 adult female ticks were sterilized in 70% ethanol for 20 min and washed three times in distilled water. Total DNA was extracted using the Qiagen DNA extraction kit (No.69506, China) according to the manufacturer’s instructions. Paired-end libraries were constructed according to the manufacturer’s instructions (Illumina) and sequenced by using the Illumina Hiseq 2500 platform.

### Data analysis

Paired-end library sequencing reads were quality controlled with FastQC (http://www.bioinformatics.babraham.ac.uk/projects/fastqc/) and trimmed on both ends using the FASTX-Toolkit, leaving high-quality nucleotides (http://hannonlab.cshl.edu/fastx_toolkit). The high-quality reads were aligned to NCBI NR databases using DIAMOND [25] with the default parameter. Then, alignment files were imported into MEGAN6[26], and the program automatically calculated a taxonomic classification of the reads. The results were interactively viewed and inspected. Multiple datasets were simultaneously opened in a single comparative document that provided comparative views of the different classifications.

Venn diagram (http://bioinformatics.psb.ugent.be/webtools/Venn/), heatmap, and phylogeny (http://itol.embl.de/help.cgi) were used to show shared phyla and genera [27] for assessing their abundance and relationship. The Shannon diversity index, Simpson's reciprocal index, and the rare fraction curve were performed by using MEGAN.

### Ethics statement

Ethics approval for this investigation was obtained from the Research Ethics Committee, Beijing Institute of Genomics, Chinese Academy of Sciences. There is no specific permission required for tick collection in the forest region of Suifenhe City (E = 131.17, N = 44.38), Heilongjiang Province of northeastern China. Our study didn’t involve any endangered or protected species.

## Results

### Experimental design and data acquisition

We divide samples into three groups: wild, direct collection from the wild; R1, reared in the laboratory as the first generation; and R8, reared as the 8^th^ generation. We use 40 and 30 adult females for DNA extraction for the wild and reared groups, respectively, since the entire animals are ground together. The sequences are from paired-end libraries and one lane for wild group, another lane for R1 and R8 group. Sequence coverage and read length are both within their standards; we note that the matched (protein sequences collected for Blastp) reads are an order of magnitude lower in R8 than the rest, both wild and R1 (Table 1).

**Table 1:** Sequence data.

Sequence library name	Wild	R1	R8
Raw data (bp)	46736012194	22457221932	24850090298
Raw reads (101 bp in average)	231366397	111174366	123020249
Matched reads	58934799	57651233	7084905

**Fig 1:**
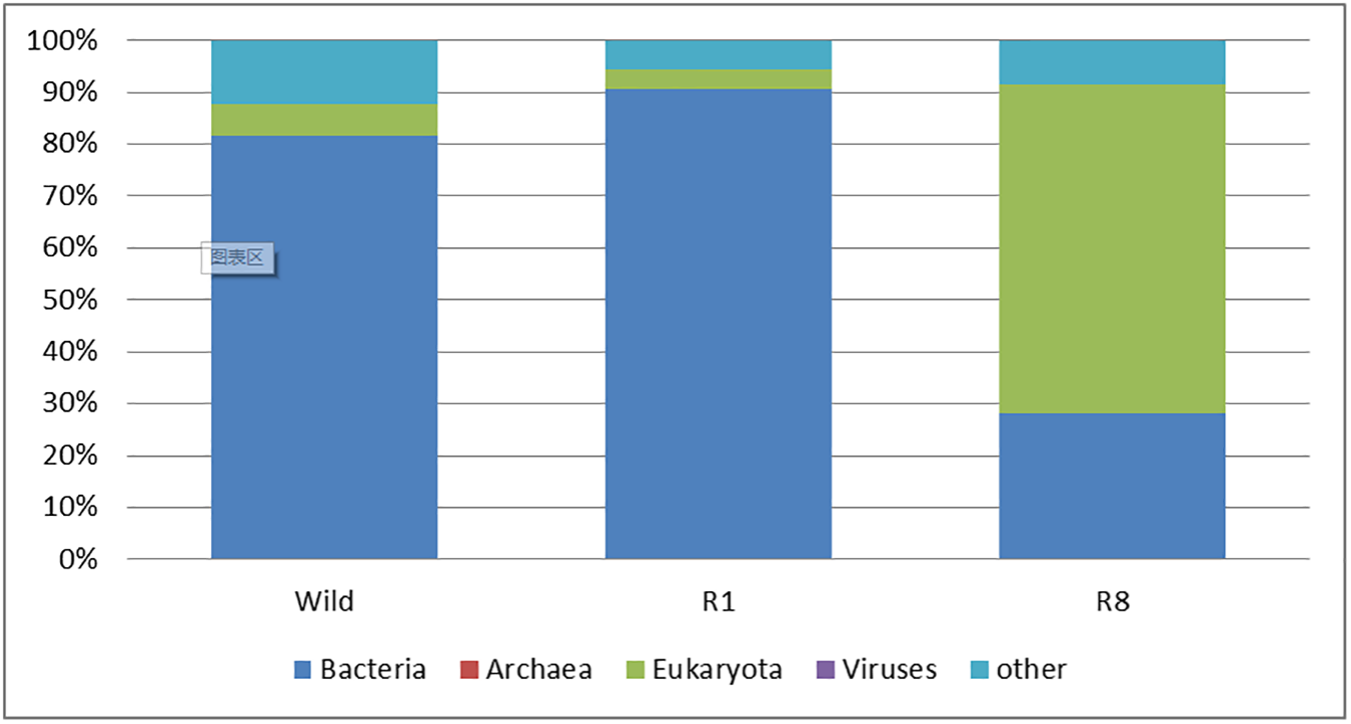
Kingdom classification of the three groups. Sequences that failed to be classified are assigned as other. Note that bacteria dominate in wild and R1 whereas eukaryotes become the greater majority in R8. Viruses are assigned in all groups but archaea are found present only in wild and R1.

Our raw data from the three groups range from 23 Gb to 47 Gb in total nucleotides and from 111 million to 231 million in sequencing reads. Annotation based on Blastp narrows useful reads into 59 million for wild, 58 million for R1, 7.1 million for R8. Rare fraction curve analysis indicates (Fig 1) data saturation ranges at the genus level: the curves for the wild, R1, and R8 ticks plateau at 45, 25, and 1 million reads, respectively. An overall reduction of the bacterial flora for the reared ticks is obvious. Next, we discuss the data at different taxonomic levels.

#### The kingdom classification

**Fig 2:**
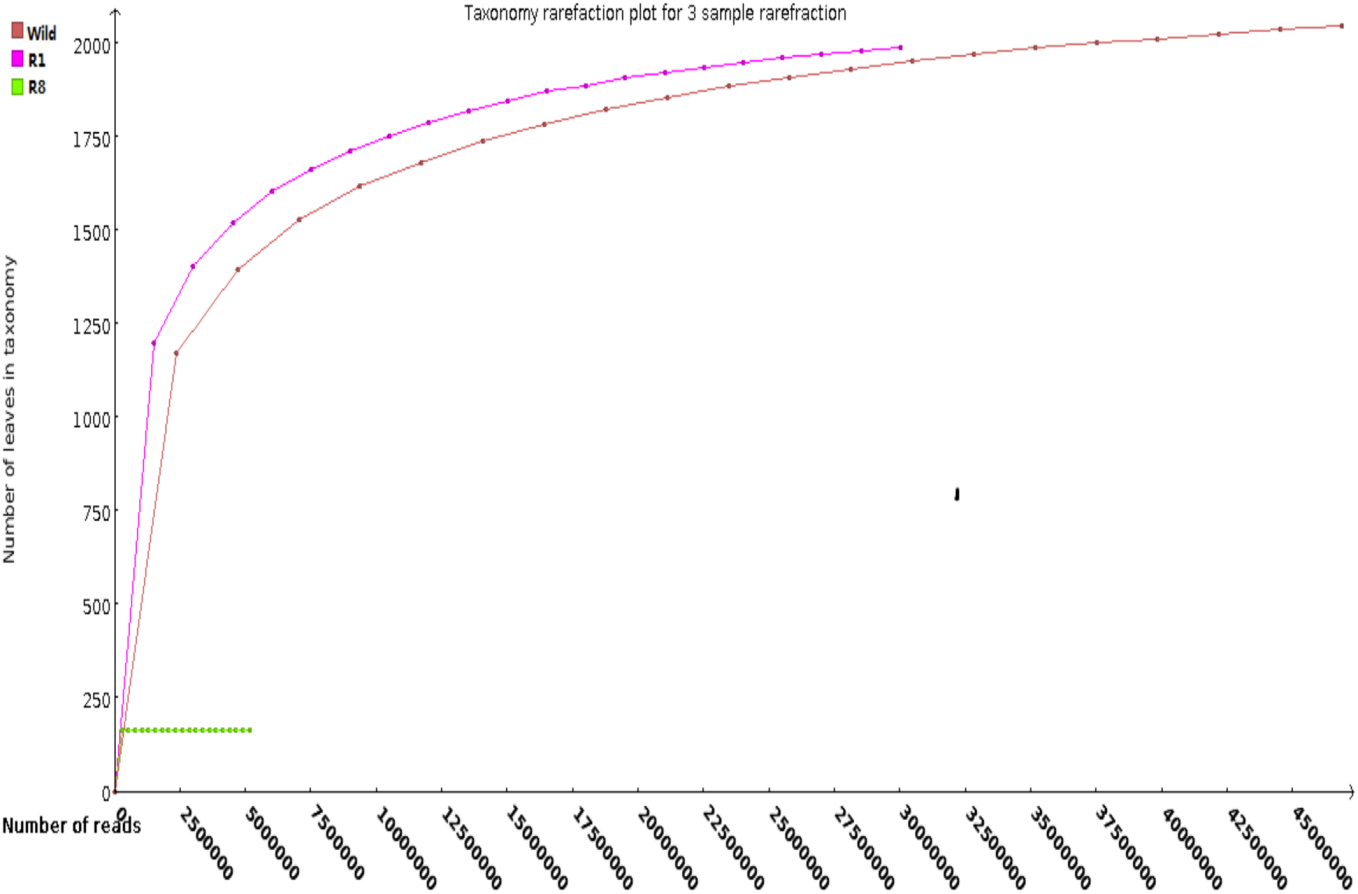
Rarefraction curves of the three tick groups at the genus level. The plot shows the number of genera assigned as the number of classified reads. The curves for wild, R1, and R8 plateau at 45, 25, and 1 million reads, respectively.

We annotate 80.39%, 89.96%, and 26.31% of all assigned bacterial reads of the groups, wild, R1, and R8, respectively, at the kingdom level (Fig 2). The annotated reads of eukaryotes show different ratios among the groups: 6.2% for wild, 3.65% for R1, and 69.57% for R8. It appears that rearing process tends to increase the fraction of eukaryotes. Archaean data exhibit another trend similar to that of the bacteria but rather unique in characteristics: 424 for wild, 2143 for R1, null for R8. Similarly, the minor component, viruses, displays its own trend: 30632 for wild, 3497 for R1, and 2036 for R8.

#### The phylum classification

**Fig 3:**
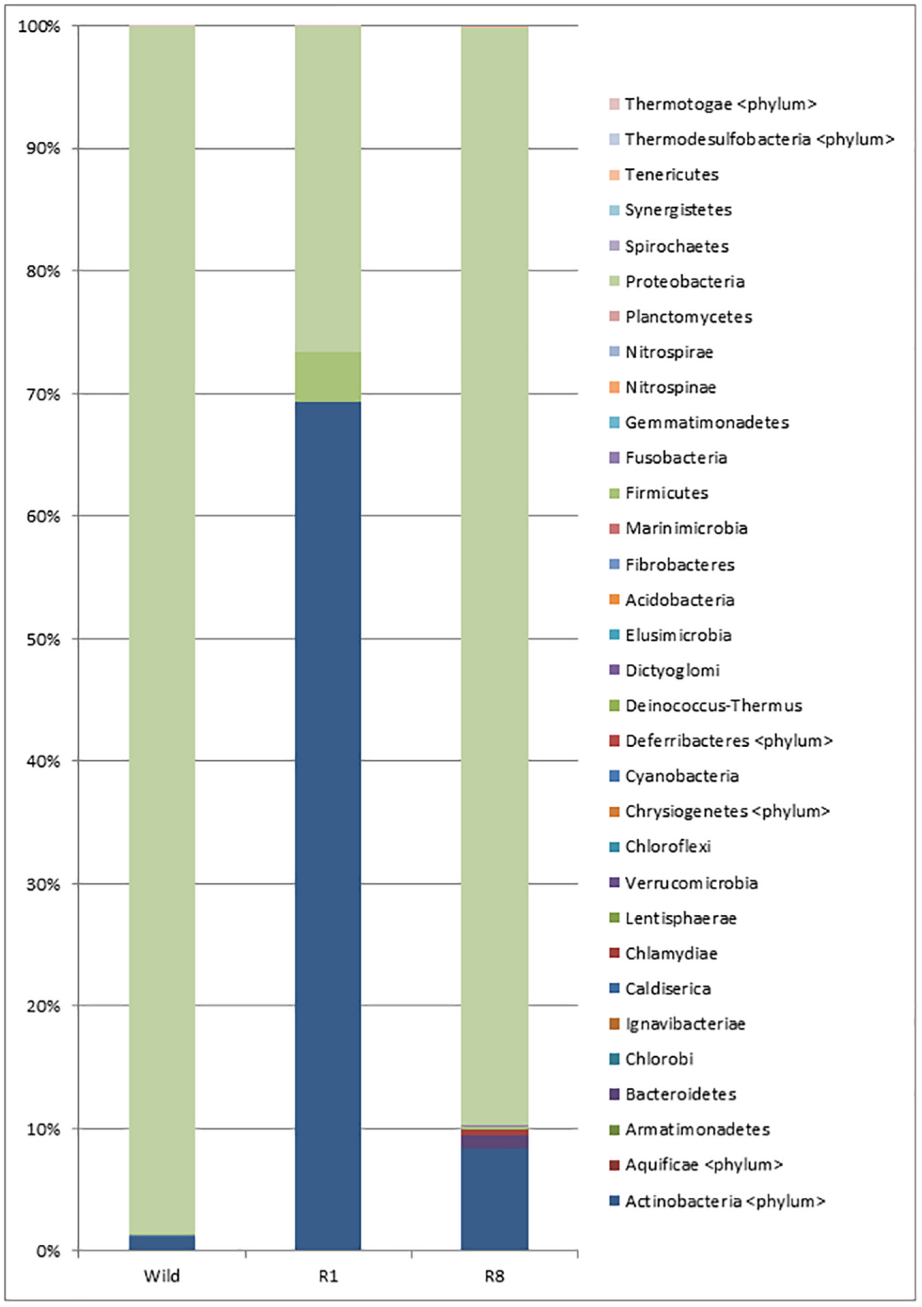
Phylum classification of the three groups. There are 29, 31 and 8 phyla in wild, R1 and R8, respectively. Phyla with a relative abundance greater than 0.1% were difined as the major phyla.

**Fig 4:**
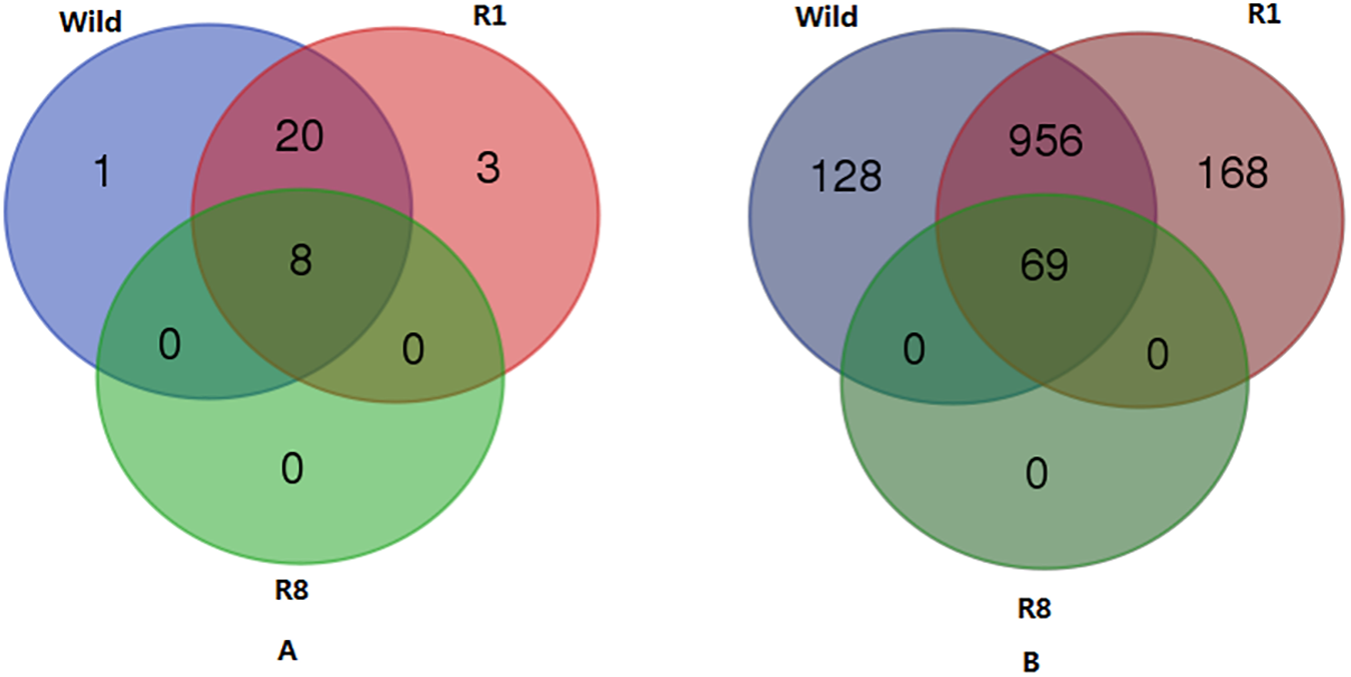
Comparison of phylum (A) and genus (B) classification among the three groups.

At the phylum level, the annotated read distribution is 29 for wild, 31 for R1, and 8 for R8, where all 8 phyla in R8 are shared by the rest, representing candidates for the core bacterial flora. Since most phyla are accounted for only a small fraction of the total reads, so we define the major bacterial phyla as those greater than 1% (Figs 3 and 4). The major phyla in the wild group are Proteobacteria and Actinobacteria. After one generation of rearing, the major R1 phyla recruit Firmicutes and Bacteroidetes. The most abundant phylum, Proteobacteria, in wild is 98.5% of the total but they become lower after rearing, 26.5% in R1, and it arises to 89.6% in R8. The most dominant phylum in R1 is Actinobacteria but it becomes much lower, 1.2% in wild, and it becomes rather moderate, 8.4% in R8.

#### The genus classification

At the genus level, there are 1153, 1193, and 69 genera for wild, R1, and R8 (Table 2), respectively. Species diversity in a community can be characterized by Shannon diversity and Simpson's indices; both account for abundance and evenness of the species present. As shown in Table 2, both indices have the same trend: wild < R8 < R1. The trend suggests that R1 may have more genera as it perserves bacteria from both wild and the early stage of rearing. The unique genera identified among the groups indicates the same result: 128 for wild, 168 for R1 and 69 for R8.

**Table 2:** Taxonomic diversity of the *I*. *persulcatus* core bacterial flora.

	Wild	R1	R8
Bacteria assigned reads (ratio)	47377256 (80.39%)	51863187 (89.96%)	1864190 (26.31%)
Bacterial genra	1153	1193	69
Shannon diversity index	2.424	3.951	3.458
Simpson's reciprocal index	2.721	8.895	4.366

#### The bacterial core flora of *I*. *persulcatus*

**Fig 5:**
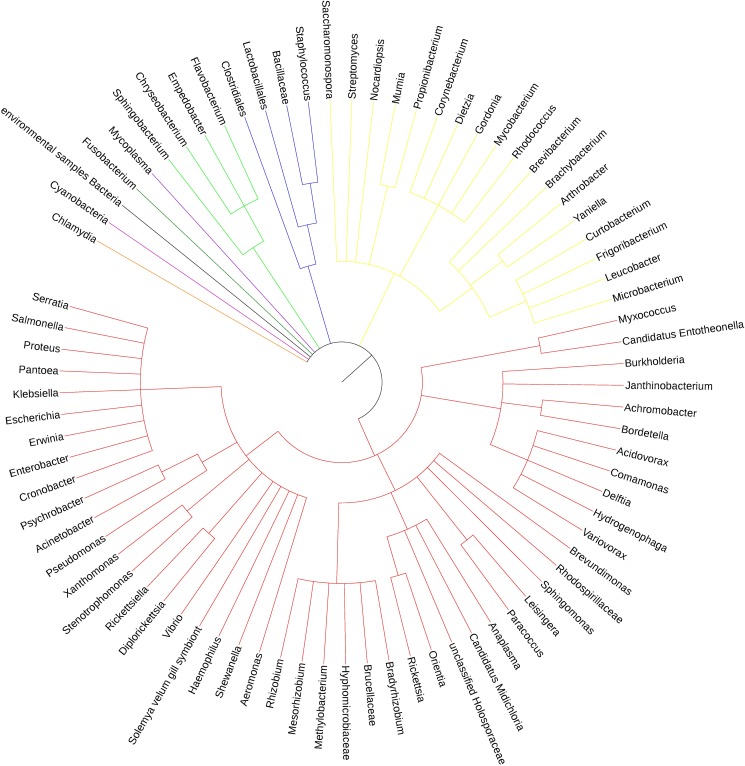
Phylogenetic tree of the shared phyla and genera by the three groups.

**Fig 6:**
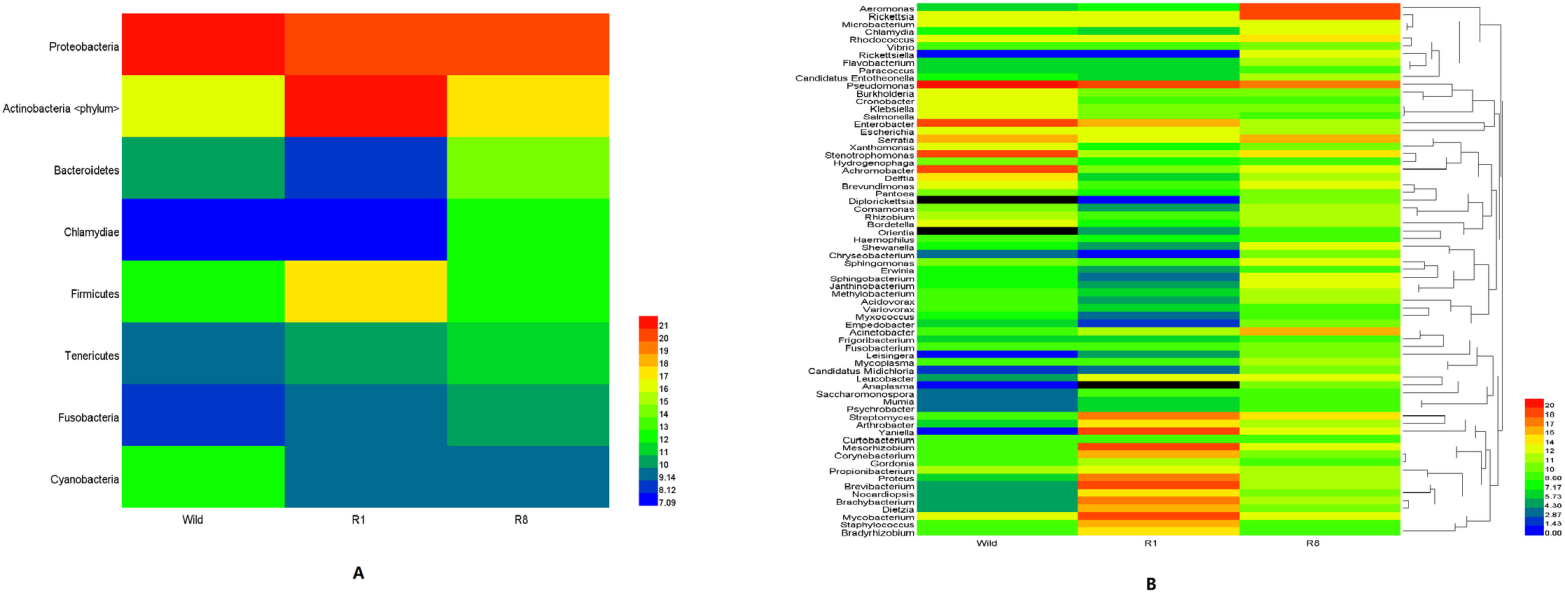
Heatmap of the shared phyla (A) and genera (B) of the three groups to highlight the abundance of taxonomic classes.

Continuous rearing in a sterile environment has lead to only 69 genera in the R8 ticks; these bacteria belong to 8 different phyla that are shared among all three groups. Therefore, we tentatively attribute the 69 genera to be the core bacterial flora of the tick. After normalization, the sequence reads of all three groups (Figs 5 and 6) are mostly Proteobacteria (43 genera), Actinobacteria (18 genera), and Bacteroidetes (4 genera); and the remaining 4 phyla each contain a single genus and Firmicutes has no genus assigned to.

At the species level, the 69 genera of the core tick bacterial flora have 1219 species for wild, 1307 species for R1, and 74 species for R8, of which 70 species are shared by both wild and R1. For example, wild has 38 species of *Rickettsia* genus, R1 has 22, and R8 has 7; the 7 species of R8 are all shared by the rest 2 groups.

## Discussion

As a predominant hard tick species in Europe, central and northern Asia, China, and Japan, *I*. *persulcatus* has been studied by using metagenomics [22, 28]. Up until 2015, a total of 16 tick metagenomics reports have been published [15], which have investigated several important parameters including species, sex, organ, and life cycle, referencing tick-borne diseases [15, 19]. However, such studies have not interrogated tick metagenomes under controlled rearing conditions that further our knowledge on tick flora and its variation under different environmental settings. Tick flora refers to its total symbionts residing mainly in the internal organs and skin since separation of these anatomic portions are not desirable at the early stage of flora definition. We also attempt to define the bacterial flora initially and to narrow the scope of the study to the core bacterial flora due to poor annotations of the eukaryotic components (such as unknow fungi and molds). In this study, we focus our attention on the bacterial communities and their diversity shift between wild ticks and those reared under SPF environment over multiple generations.

In our study, concerning methodology, we took one of the two popular approaches, genome-wide sequencing approach rather than transcriptomes sequencing, and hope to provide an initial definition of the tick core bacterial flora. Previous reports on tick metagenomics have not used genome-wide sequencing approach [15] and they have mostly used 16s rRNA-based methodology [22], having sampled 16s rRNA of wild ticks and blood meal ticks. Taking a genome-wide sequencing approach, however, we are limited by the number of samples that can be handled for an initial analysis, and we therefore only examined three data points at such a stage. More detailed sampling certainly necessary but a careful design of such a experiment is of essence. For instance, it appears that at the 8^th^ generation the core bacterial flora becomes stabilized. We also need to know how stable such floras are for ticks from a single origin and how variable it can be for ticks from different origins, even at the individual level in an ultimate sense. There are, of course, more questions can be asked for much more details as to how the floras change gradually from the wild conditions to the rearing in each generation, especially under different controlled rearing, such as pathogen-free vs natural hosts. Ample categorization procedures are of importance for more systematic studies.

Nevertheless, such a study is already very informative for further definition of the core floras of the tick under various conditions. First, we notice that at the kingdom level R8 has much less annotated reads as compared to the rest two samples. Judging by the increase of eukaryotic reads, it is reasonable to attribute the reduced annotation to unknown eukaryotes. Another loss in R8 is Archaea, similar to the case of eukaryotes. Second, the dramatic reduction of bacterial phyla and genera after rearing suggests the fact that most of the tick dwelling microbes from the wild are both temporary and environment-associated. It is obvious that an effort to define a species-associated core bacterial flora is both necessary and important for *I*. *persulcatus*. Third, we have identified a tentative species-associated core bacterial flora that is composed of 69 genera and 70 species. We notice that the number of species is almost equal to that of the genera as the tick core bacterial flora, and such a phenomenon is rather common due to relative less certainty at the species level. Fourth, the largest number of species common in all 3 experimental groups are from the genus *Rickettsia*, which are endosymbiont of *I*. *scapularis*. This species plays an important role in sex determination in ticks [29].

High-throughput sequencing, coupled with bioinformatics analysis, provides a powerful tool for defining microbiome or flora of ticks, and such floras and their variations provide diagnostic markers or clues for disease transmission and infection mechanisms. Our results represent initial steps that uncover the species-associated flora and its variability under the influence of environmental elements, including hosts, geography, climates, and etc. Our results justify further metagenomic studies on the tick, including further anatomic stratifications.

## References

[1] Fuente JDL, Blouin EF, Kocan KM. Infection Exclusion of the Rickettsial Pathogen Anaplasma marginale in the Tick Vector Dermacentor variabilis. Clinical & Diagnostic Laboratory Immunology. 2003;10(1):182–4. 12522060 10.1128/CDLI.10.1.182-184.2003 PMC145288

[2] Jääskeläiinen Anu E, Tonteri Elina, Sironen Tarja, et al European subtype tick-borne encephalitis virus in Ixodes persulcatus ticks. Emerging Infectious Diseases. 2011;17(2):323–5. doi: 10.3201/eid1702.101487 21291624 10.3201/eid1702.101487 PMC3376769

[3] Chengxu AI, Renjie HU, Hyland KE, Wen Y, Zhang Y, Qiu Q, et al Epidemiological and aetiological evidence for transmission of Lyme disease by adult Ixodes persulcatus in an endemic area in China. 1991;19(4):1061–5. 10.1093/ije/19.4.1061 2083990

[4] Cao WC, Zhao QM, Zhang PH, Dumler JS, Zhang XT, Fang LQ, et al Granulocytic Ehrlichiae in Ixodes persulcatus Ticks from an Area in China Where Lyme Disease Is Endemic. Journal of Clinical Microbiology. 2000;38(11):4208–10. 11060091 10.1128/jcm.38.11.4208-4210.2000 PMC87564

[5] Cao WC, Zhao QM, Zhang PH, Yang H, Wu XM, Wen BH, et al Prevalence of Anaplasma phagocytophila and Borrelia burgdorferi in Ixodes persulcatus ticks from northeastern China. American Journal of Tropical Medicine & Hygiene. 2003;68(5):547–50. 12812342 10.4269/ajtmh.2003.68.547

[6] Sun Y, Liu G, Yang L, Xu R, Cao W. Babesia microti-like rodent parasites isolated from Ixodes persulcatus (Acari: Ixodidae) in Heilongjiang Province, China. Veterinary parasitology. 2008;156(3–4):333–9. doi: 10.1016/j.vetpar.2008.05.026 18718720 10.1016/j.vetpar.2008.05.026

[7] Niu Q, Liu Z, Yang J, Yu P, Pan Y, Zhai B, et al Genetic diversity and molecular characterization of Babesia motasi-like in small ruminants and ixodid ticks from China. Infection Genetics & Evolution Journal of Molecular Epidemiology & Evolutionary Genetics in Infectious Diseases. 2016;41:8–15. 10.1016/j.meegid.2016.03.007 26976477

[8] Jia N, Jiang JF, Huo QB, Jiang BG, Cao WC. Rickettsia sibirica subspecies sibirica BJ-90 as a cause of human disease. The New England journal of medicine. 2013;369(12):1176–8. doi: 10.1056/NEJMc1303625 . 24047079 10.1056/NEJMc1303625

[9] Sun J, Lin J, Gong Z, Chang Y, Ye X, Gu S, et al Detection of spotted fever group Rickettsiae in ticks from Zhejiang Province, China. Experimental & applied acarology. 2015;65(3):403–11. doi: 10.1007/s10493-015-9880-9 ; PubMed Central PMCID: PMC4322220. 25633265 10.1007/s10493-015-9880-9 PMC4322220

[10] Consortium HMJRS, Nelson KE, Weinstock GM, Highlander SK, Worley KC, Creasy HH, et al A catalog of reference genomes from the human microbiome. Science. 2010;328(5981):994–9. doi: 10.1126/science.1183605 20489017 10.1126/science.1183605 PMC2940224

[11] Ottman N, Smidt H, de Vos WM, Belzer C. The function of our microbiota: who is out there and what do they do? Frontiers in cellular and infection microbiology. 2012;2:104 doi: 10.3389/fcimb.2012.00104 ; PubMed Central PMCID: PMC3417542. 22919693 10.3389/fcimb.2012.00104 PMC3417542

[12] Oulas A, Pavloudi C, Polymenakou P, Pavlopoulos GA, Papanikolaou N, Kotoulas G, et al Metagenomics: tools and insights for analyzing next-generation sequencing data derived from biodiversity studies. Bioinformatics and biology insights. 2015;9:75–88. doi: 10.4137/BBI.S12462 ; PubMed Central PMCID: PMC4426941. 25983555 10.4137/BBI.S12462 PMC4426941

[13] Faust K, Lahti L, Gonze D, de Vos WM, Raes J. Metagenomics meets time series analysis: unraveling microbial community dynamics. Current Opinion in Microbiology. 2015;25:56–66. doi: 10.1016/j.mib.2015.04.004 26005845 10.1016/j.mib.2015.04.004

[14] Aguiar-Pulido V, Huang W, Suarez-Ulloa V, Cickovski T, Mathee K, Narasimhan G. Metagenomics, Metatranscriptomics, and Metabolomics Approaches for Microbiome Analysis. Evolutionary bioinformatics online. 2016;12(Suppl 1):5–16. doi: 10.4137/EBO.S36436 ; PubMed Central PMCID: PMC4869604. 27199545 10.4137/EBO.S36436 PMC4869604

[15] Narasimhan S, Fikrig E. Tick microbiome: the force within. Trends in parasitology. 2015;31(7):315–23. doi: 10.1016/j.pt.2015.03.010 ; PubMed Central PMCID: PMC4492851. 25936226 10.1016/j.pt.2015.03.010 PMC4492851

[16] Williams-Newkirk AJ, Rowe LA, Mixson-Hayden TR, Dasch GA. Characterization of the bacterial communities of life stages of free living lone star ticks (Amblyomma americanum). PloS one. 2014;9(7):e102130 doi: 10.1371/journal.pone.0102130 ; PubMed Central PMCID: PMC4108322. 25054227 10.1371/journal.pone.0102130 PMC4108322

[17] Papa A, Tsioka K, Kontana A, Papadopoulos C, Giadinis N. Bacterial pathogens and endosymbionts in ticks. Ticks and tick-borne diseases. 2017;8(1):31–5. doi: 10.1016/j.ttbdis.2016.09.011 . 27686386 10.1016/j.ttbdis.2016.09.011

[18] Ponnusamy Loganathan aAG, b Van Treuren Will,b Weiss Sophie,c Parobek Christian M.,d Juliano Jonathan J.,e, Knight Rob b, f Roe R. Michael,a Apperson Charles S.,a,g Meshnickh Steven R. Diversity of Rickettsiales in the Microbiome of the Lone Star Tick, Amblyomma americanum. Applied and environmental microbiology. 2013;80 doi: 10.1128/AEM.02987-13 24162580 10.1128/AEM.02987-13 PMC3910995

[19] Porter L, Radulovic Z, Kim T, Braz GR, Da Silva Vaz I Jr., Mulenga A. Bioinformatic analyses of male and female Amblyomma americanum tick expressed serine protease inhibitors (serpins). Ticks and tick-borne diseases. 2015;6(1):16–30. doi: 10.1016/j.ttbdis.2014.08.002 ; PubMed Central PMCID: PMC4252504. 25238688 10.1016/j.ttbdis.2014.08.002 PMC4252504

[20] van Overbeek L, Gassner F, van der Plas CL, Kastelein P, Nunes-da Rocha U, Takken W. Diversity of Ixodes ricinus tick-associated bacterial communities from different forests. FEMS microbiology ecology. 2008;66(1):72–84. doi: 10.1111/j.1574-6941.2008.00468.x . 18355299 10.1111/j.1574-6941.2008.00468.x

[21] Igolkina Y, Bondarenko E, Rar V, Epikhina T, Vysochina N, Pukhovskaya N, et al Genetic variability of Rickettsia spp. in Ixodes persulcatus ticks from continental and island areas of the Russian Far East. Ticks and tick-borne diseases. 2016;7(6):1284–9. doi: 10.1016/j.ttbdis.2016.06.005 . 27424272 10.1016/j.ttbdis.2016.06.005

[22] Zhang XC, Yang ZN, Lu B, Ma XF, Zhang CX, Xu HJ. The composition and transmission of microbiome in hard tick, Ixodes persulcatus, during blood meal. Ticks and tick-borne diseases. 2014;5(6):864–70. doi: 10.1016/j.ttbdis.2014.07.009 . 25150725 10.1016/j.ttbdis.2014.07.009

[23] Bikel S, Valdez-Lara A, Cornejo-Granados F, Rico K, Canizales-Quinteros S, Soberon X, et al Combining metagenomics, metatranscriptomics and viromics to explore novel microbial interactions: towards a systems-level understanding of human microbiome. Computational and structural biotechnology journal. 2015;13:390–401. doi: 10.1016/j.csbj.2015.06.001 ; PubMed Central PMCID: PMC4484546. 26137199 10.1016/j.csbj.2015.06.001 PMC4484546

[24] Turaev D, Rattei T. High definition for systems biology of microbial communities: metagenomics gets genome-centric and strain-resolved. Current opinion in biotechnology. 2016;39:174–81. doi: 10.1016/j.copbio.2016.04.011 . 27115497 10.1016/j.copbio.2016.04.011

[25] Buchfink B, Xie C, Huson DH. Fast and sensitive protein alignment using DIAMOND. Nature methods. 2014;12(1):59 doi: 10.1038/nmeth.3176 25402007 10.1038/nmeth.3176

[26] Huson DH, Beier S, Flade I, Górska A, Elhadidi M, Mitra S, et al MEGAN Community Edition—Interactive Exploration and Analysis of Large-Scale Microbiome Sequencing Data. PLoS computational biology. 2016;12(6):e1004957 doi: 10.1371/journal.pcbi.1004957 27327495 10.1371/journal.pcbi.1004957 PMC4915700

[27] Deng W, Wang Y, Liu Z, Cheng H, Xue Y. HemI: a toolkit for illustrating heatmaps. PloS one. 2014;9(11):e111988 doi: 10.1371/journal.pone.0111988 25372567 10.1371/journal.pone.0111988 PMC4221433

[28] Kurilshikov A, Livanova NN, Fomenko NV, Tupikin AE, Rar VA, Kabilov MR, et al Comparative Metagenomic Profiling of Symbiotic Bacterial Communities Associated with Ixodes persulcatus, Ixodes pavlovskyi and Dermacentor reticulatus Ticks. PloS one. 2015;10(7):e0131413 doi: 10.1371/journal.pone.0131413 ; PubMed Central PMCID: PMC4496043. 26154300 10.1371/journal.pone.0131413 PMC4496043

[29] Kageyama D, Narita S, Watanabe M. Insect Sex Determination Manipulated by Their Endosymbionts: Incidences, Mechanisms and Implications. Insects. 2012;3(1):161–99. doi: 10.3390/insects3010161 ; PubMed Central PMCID: PMC4553623. 26467955 10.3390/insects3010161 PMC4553623

